# History of adverse pregnancy outcomes and cognitive decline among older adults: the Adult Changes in Thought study

**DOI:** 10.1002/alz.089330

**Published:** 2025-01-09

**Authors:** Sarah E. Tom, Laura B Harrington, Kristi Chau, Paige D Wartko, Roxanne Muiruri, Chloe Krakauer, Linda K. McEvoy, Andrea Z. LaCroix, Eliza C. Miller

**Affiliations:** ^1^ Columbia University Irving Medical Center, New York, NY USA; ^2^ University of Washington, Seattle, WA USA; ^3^ Kaiser Permanente Washington Health Research Institute, Seattle, WA USA; ^4^ Kaiser Permanente Bernard J. Tyson School of Medicine, Pasadena, CA USA; ^5^ Columbia University, New York, NY USA; ^6^ 3Kaiser Permanente Washington Health Research Institute, Seattle, WA USA; ^7^ Kaiser Permanente Washington, Seattle, WA USA; ^8^ Kaiser Permanente Washington Research Institute, Seattle, WA USA; ^9^ University of California, San Diego, La Jolla, CA USA; ^10^ University of California San Diego, La Jolla, CA USA

## Abstract

**Background:**

Whether history of adverse pregnancy outcomes (APOs) contributes to cognitive decline in later life is not understood because few cohorts have information on pregnancy history and later‐life cognition. We hypothesized that history of any APO would be related to lower global cognition and faster cognitive decline among older adults, compared to no APO history.

**Method:**

The Adult Changes in Thought (ACT) Study is a prospective cohort study embedded in an integrated health care system of adults aged 65 and older without dementia at enrollment. Among ACT participants enrolled between 1994 and 2020 (n = 5,763, 58% women), women eligible for our study were born in 1940 or later, had evidence of ≥1 child or pregnancy, and had maternal or pregnancy information in their medical record (n = 444). APOs of interest were preterm birth, hypertensive disorders of pregnancy (including gestational hypertension and preeclampsia), low birth weight, small for gestational age, and stillbirth. Cognition at ACT enrollment and every two years thereafter was assessed by the Cognitive Abilities Screening Instrument, a 40‐item instrument of global cognition, further processed using item‐response theory (CASI‐IRT); points are standard deviation units of CASI‐IRT in the study population. We fit linear models using generalized estimating equations to assess associations between history of any APO with (1) cognition score and (2) cognitive decline.

**Result:**

Among eligible participants, 13% had a history of any APO. Among people of the same age, birth cohort, education, and marital status, CASI‐IRT scores were expected to be 0.23 point lower (95% CI: ‐0.54, 0.07) among people with a history of APOs than those without; this difference did not reach statistical significance. Expected 4‐year rates of cognitive change among people with a history of APOs [‐0.01; 95% CI ‐0.25, 0.23] was not found to differ significantly from those without [0.02; 95% CI ‐0.05, 0.09].

**Conclusion:**

There is some inconclusive evidence that history of any APO may be associated with lower cognition level in older adulthood. We failed to find any convincing evidence of the association between history of APOs and the rate of cognitive decline.

